# Cytoplasmic accumulation of NCoR in malignant melanoma: consequences of altered gene repression and prognostic significance

**DOI:** 10.18632/oncotarget.3252

**Published:** 2015-03-19

**Authors:** Fernando Gallardo, Andreina Padrón, Ricard Garcia-Carbonell, Cristina Rius, Abel González-Perez, Montserrat Arumí-Uria, Mar Iglesias, Lara Nonell, Beatriz Bellosillo, Sonia Segura, Ramon Maria Pujol, Nuria Lopez-Bigas, Joan Bertran, Anna Bigas, Lluís Espinosa

**Affiliations:** ^1^ Dermatology Department, Parc de Salut Mar-Hospital del Mar, Barcelona, Spain; ^2^ Pathology Department, Parc de Salut Mar-Hospital del Mar, Barcelona, Spain; ^3^ Stem Cells and Cancer Research Laboratory, Institut Hospital del Mar Investigacions Mèdiques (IMIM), Barcelona, Spain; ^4^ Research Unit on Biomedical Informatics, Department of Experimental and Health Sciences, Universitat Pompeu Fabra, Barcelona, Spain; ^5^ Servei d’Anàlisi de Microarrays, Institut Hospital del Mar Investigacions Mèdiques (IMIM), Barcelona, Spain; ^6^ Catalan Institution for Research and Advanced Studies (ICREA), Passeig Lluís Companys, Barcelona, Spain; ^7^ Universitat de Vic, Universitat Central de Catalunya (UVic-UCC), Vic, Spain

**Keywords:** NCoR, melanoma progression, IKK, gene transcription

## Abstract

Invasive malignant melanoma (MM) is an aggressive tumor with no curative therapy available in advanced stages. Nuclear corepressor (NCoR) is an essential regulator of gene transcription, and its function has been found deregulated in different types of cancer. In colorectal cancer cells, loss of nuclear NCoR is induced by Inhibitor of kappa B kinase (IKK) through the phosphorylation of specific serine residues. We here investigate whether NCoR function impacts in MM, which might have important diagnostic and prognostic significance. By IHC, we here determined the subcellular distribution of NCoR in a cohort of 63 primary invasive MM samples, and analyzed its possible correlation with specific clinical parameters. We therefore used a microarray-based strategy to determine global gene expression differences in samples with similar tumor stage, which differ in the presence of cytoplasmic or nuclear NCoR. We found that loss of nuclear NCoR results in upregulation of a specific cancer-related genetic signature, and is significantly associated with MM progression. Inhibition of IKK activity in melanoma cells reverts NCoR nuclear distribution and specific NCoR-regulated gene transcription. Analysis of public database demonstrated that inactivating NCoR mutations are highly prevalent in MM, showing features of driver oncogene.

## INTRODUCTION

Malignant melanoma (MM) is the most aggressive form of skin cancer accounting for most skin cancer deaths. Worldwide raising MM incidences have been recorded in the last decades, and currently about 48,000 melanoma-related deaths occur every year. MM behavior mainly depends on the stage of disease but it varies from patient to patient, likely reflecting differences in the biological features of the tumor.

Three main molecular pathways are found invariably deregulated in melanocytic tumors, including the RAS-RAF-MEK-ERK pathway (through mutation of *BRAF*, *NRAS* or *KIT*), the p16INK4A-CDK4-RB pathway (through mutation of *INK4A* or *CDK4*), and the ARF-p53 pathway (through mutation of *ARF* or *TP53*). Less frequently, other pathways such as the PI3K-AKT pathway (through mutation of *NRAS*, *PTEN* or *PIK3CA*) and the canonical Wnt signaling pathway (through mutation of *CTNNB1* or *APC genes*) have been also associated with MM [1]. The RAS/RAF/MEK/ERK pathway is a crucial regulator of proliferation and cell survival in different physiologic and pathologic systems, and it is supposed to contribute to tumor progression in part through the activation of other downstream pathways such as NF-κB [2].

The NF-κB transcription factor is a homo- or hetero-dimeric factor involving five different proteins (p50, p52, p65, c-Rel, and RelB), which plays an essential role in inflammation but also in the regulation of specific cellular functions such as apoptosis, proliferation, cell migration and metastasis. In the absence of external stimuli, NF-κB factors are mostly localized in the cytoplasm bound to the inhibitor of kappa B (IκB) proteins. Phosphorylation of IκB by the IκB kinase (IKK) complex leads to IκB ubiquitination and degradation, resulting in the release of NF-κB that then translocates to the nucleus to activate specific transcription. Thus, detection of nuclear NF-κB in both normal or neoplastic cells is indicative of NF-κB and IKK activation. Constitutive NF-κB/IKK activation has been previously found in different types of cancer including MM [3]. NF-*κ*B plays an important role in preventing tumor cell apoptosis through the induction of anti-apoptotic genes such as the Inhibitors of Apoptosis (IAP) c-IAP1 and c-IAP2, or the melanoma inhibitor of apoptosis (ML-IAP) [4]. NF-*κ*B also controls the expression of several chemokines, interleukin IL-1 and IL-6, the vascular endothelial growth factor (VEGF), as well as other factors that are known to impact in MM progression [4].

Besides their role in NF-*κ*B activation, the IKK kinases regulate other substrates that are directly involved in the control of gene expression such as the silencing mediator of retinoic and thyroid hormone receptors (SMRT) and the nuclear receptor corepressor (NCoR). Both proteins are components of specific multi-protein complexes that contain histone deacetylases (HDACs) and act as critical regulators of gene repression. Alterations in the repressive function of HDAC and NCoR have been found associated to different types of cancer, although little is known on how their deregulation impacts in MM.

Because the IKK kinases promote the exportation of NCoR (cNCoR) and SMRT [5, 6] corepressors to cytoplasm in colorectal cancer (CRC) cells, we speculated that IKK activity might also affect NCoR distribution and specific gene transcription in MM, and tumor behavior. In this study, we have determined the subcellular distribution of NCoR, and the presence of nuclear p65-NF-κB as a surrogate marker of IKK complex activation, in a set of human MM samples and melanocytic benign nevi. In addition, we have performed a large-scale gene expression analysis of MM samples with different levels of nuclear NCoR (nNCoR). Our results provide molecular information for the classification of MM subtypes, but also for prognostic and stratification of patients that could benefit for future personalized therapies.

## RESULTS

### NCoR shows a heterogeneous subcellular distribution in MM samples

**Figure 1:**
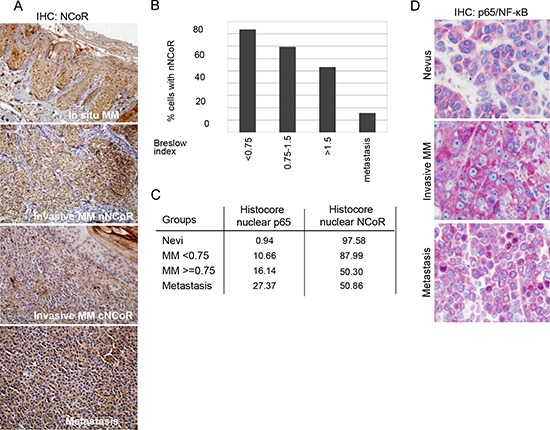
Loss of nuclear NCoR is associated with MM progression **(A)** IHC analysis of NCoR distribution in MM samples at different stages of tumor progression. **(B)** Quantification of the percent of cells containing detectable nuclear NCoR in tumors with different Breslow Index. **(C)** Histoscore index showing the proportion of cells carrying nuclear (active) p65-NF-κB and nuclear NCoR in the indicated MM groups. **(D)** IHC analysis of p65-NF-κB distribution in nevi and MM samples at different stages of tumor progression.

By IHC, we found that NCoR protein exhibits a nuclear distribution in melanocytes of nevi samples. However, malignant melanocytes in MM samples showed a variable distribution, which in some cases resulted in the loss of nNCoR, occasionally associated with its cytoplasmic accumulation. More specifically, we observed that all *in situ* MM samples contained nNCoR similar to benign nevi. In contrast, the number of cells that lost nNCoR at the time of diagnosis correlated with increased tumor staging (Figure 1A and 1B), whereas the vast majority of metastatic samples disclosed an absent or minimal number of melanocytic cells with nNCoR. To determine the predictive value of loss of nNCoR in the primary tumors, we analyzed NCoR distribution in 63 primary tumor samples from MM patients with different Breslow index using the non-parametric Spearman test. We found an inverse correlation (–0.628) between the percentages of nNCoR and the Breslow index (*p* < 0.001), with samples showing lower percentage of nNCoR positive cells corresponding to the greater Breslow index (Figure 1B, Table 1). Next, we analyzed the possible relationship between loss of nNCoR and other prognostic indicators of MM. The analysis demonstrated a significant association between loss of nNCoR localization and higher mitotic index (*p* < 0.01) and a statistical trend with ulceration (*p* = 0.051) (see Table 1). However, no differences in other histopathological features (regression or prevalence of inflammatory component), age and gender were recorded between MM with nNCoR or cNCoR. Moreover, we did not detect any significant association between BRAF mutational status and NCoR distribution in 18 samples analyzed (all samples with available material).

**Table 1:** **Correlation between loss of nuclear NCoR and the different prognostic clinicopathological variables.** Disease stage indicates the maximum stage reached at the end of the study.

Variable (*n*)	nuclear NCoR % cells median [P_25-_P_75_]	*p*-value
Stage		
I–II (42)	70 [56–85]	
		0.001
III–IV (21)	45 [42–53]	
Breslow index	ρ = −0.628*	<0.001
Mitotic index	ρ = −0.546*	<0.001
Nuclear p65 detection		
Moderate/Intense	ρ = −0.551*	0.027
Ulceration		
Yes (14)	45 [42–69]	
		0.051
No (49)	70 [51–82]	
Clark level		
I–III (49)	70 [58–85]	
		<0.001
IV–V (14)	45 [39–52]	
Regression features		
No (40)	70 [45–85]	
<50% (12)	60 [45–69]	0.675
> = 50% (11)	62 [50–78]	
Tumor infiltrating lymphocytes**		
TILs not identified/		
Non Brisk (36)	70 [46–83]	
		0.256
TILs Brisk (27)	56 [44–80]	

* Relationship assessed with Spearman's Rho correlation coefficient

** TILs Not Identified: No lymphocytes present, or lymphocytes present but do not infiltrate tumor at all. TILs Non Brisk: Lymphocytes infiltrate melanoma only focally or not along the entire base of the vertical growth phase. TILs Brisk: Lymphocytes diffusely infiltrate the entire base of the vertical growth phase or the entire invasive component of the melanoma

**Figure 2:**
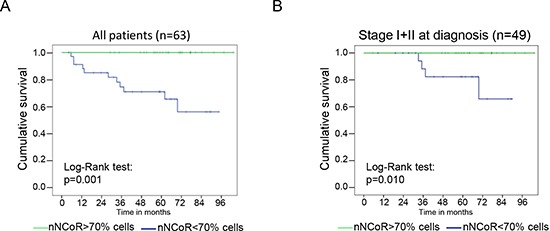
NCoR distribution predicts MM patient survival Kaplan Meier analysis of the accumulated mortality by melanoma in the whole group of study **(A)** or in the subset of patients from stage I and II of the disease **(B)**. The green line represent patients with tumors containing more than 70% of cells with nuclear NCoR, whereas the blue line represent tumors with less than 70% of cells with nuclear NCoR.

Accumulation of cNCoR showed a correlation of 0.551 with p65 nuclear distribution (indicative of IKK activation) (Figure 1C and 1D), which associated with MM progression. To further validate the relevance of NCoR localization as a predictive biomarker, patients were followed for a median of 59.4 months and analyzed based on the presence of nNCoR. Mortality rate from all causes was significantly higher in those cases with less than 70% of cells containing nNCoR (from now on cNCoR) compared with tumors maintaining nNCoR in more than 70% of cells (from now on nNCoR), with overall survival (OS) at 5 years of 60.8% and 96% respectively. The OS curves of each group, considering a cut-off above and below 70% of cells carrying nNCoR, were significantly different between both groups with a log rank of 14.626 and *p* < 0.001 (not depicted). Most importantly, the MM specific mortality (DSS) at 5 years was 29% in the cNCoR group compared to 0% in the nNCoR group (log rank 11.568, *p* = 0.001) (Figure 2A). Considering those patients with skin limited disease at diagnosis (*n* = 49) significant differences in DSS were maintained (*p* = 0.010) between nNCoR and cNCoR (Figure 2B). Disease free survival (DFS) in the cNCoR group was of 67.1% at 3 years and 58% at 5 years while no events were recorded in the nNCoR group (not depicted).

### MM show different gene expression profiles depending on NCoR localization

To study the molecular bases that support the different behavior of patients carrying cNCoR and nNCoR tumors, we performed microarray expression analysis of invasive MM tumors with similar clinicopathologic characteristics and comparable mutational status for BRAF, NRAS and KIT (Table 2), which differ in the presence of cytoplasmic (*n* = 3) or nuclear (*n* = 4) NCoR. We included in the analysis 3 benign nevi cases in which NCoR was exclusively located in the nucleus. To identify transcripts significantly altered in tumors with nNCoR or cNCoR we fixed a minimum arbitrary fold change of 1.2 and a *p*-value ≤ 0.05 as the threshold cut-off (submitted to GEO: GSE56494).

**Table 2:** Clinicopathologic characteristics and mutational status of the samples included in the expression microarray analysis (NM, nodular melanoma; SSM, superficial spreading melanoma; WT, wild type)

SAMPLE	BRAF	NRAS	KIT	MM subtype	Location	Breslow (mm)
cNCoR	WT	Q61L	WT	NM	Arm	9.0
cNCoR	V600R	WT	WT	SSM	Arm	5.3
cNCoR	WT	Q61R	WT	SSM	Trunk	4.1
nNCoR	V600E	WT	WT	SSM	Leg	4.4
nNCoR	V600E	WT	WT	NM	Trunk	8.0
nNCoR	WT	Q61L	WT	NM	Trunk	5.1
nNCoR	V600R	WT	WT	SSM	Trunk	4.7
Nevus	V600E	WT	WT			
Nevus	V600E	WT	WT			
Nevus	V600E	WT	WT			

**Figure 3:**
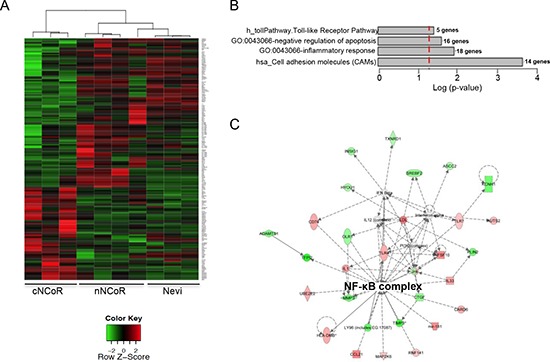
Gene expression analysis of the two MM groups and non-malignant nevi **(A)** Heatmap showing the results of the supervised analysis from genes differentially expressed between nNCoR and cNCoR cases. The list of genes appearing in the heatmap is detailed in Supplementary Table S1. **(B)** Selected Gene Ontology (Biological Processes) terms, KEGG (Kyoto Encyclopedia of Genes and Genomes) and Biocarta Pathways (http://www.biocarta.com/) obtained after a functional analysis in DAVID (http://david.abcc.ncifcrf.gov/summary.jsp). **(C)** Top network as shown by Ingenuity Pathway Analysis (http://www.ingenuity.com/). Genes in green represent those overexpressed in nNCoR and in red overexpressed in cNCoR group.

An initial analysis of global expression profiles demonstrate that whereas nevi samples clustered together in the Principal Component Analysis space, MM samples segregate randomly, and do not properly differentiate between nNCoR and cNCoR tumors (not depicted). However, supervised analysis of the data identified a 185-gene signature that clearly segregated cNCoR from nNCoR in our cohort of MM samples. Interestingly, nNCoR MM samples showed gene expression profiles comparable to the benign melanocytic nevi controls (Figure 3A). To identify putative targets of NCoR repression that are upregulated during MM progression, we focused on those transcripts that were significantly over-represented in the cNCoR group compared to the other groups. A total of 73 genes were significantly upregulated in these samples including a subset of known cancer-related genes such as IL8 [7], the heat shock proteins HSPA1A/HSPA1B* and HSPA6 [8], the tumor suppressor TFPI2, SEMA3E that is involved in tissue development and cell adhesion [9], the cell cycle regulator MAGEC2/MAGEC3 [10], EYA4 which expression is altered in hepatocellular cacinoma, the protease inhibitor and tumor promoter Serpine 1 [11, 12], the matrix metaloprotease inhibitor TIMP3 [13], and the melanoma cell adhesion molecule MCAM [14] (Supplementary Table S1).

Functional analysis of an extended list of 654 genes (*p* < 0.05) performed in Ingenuity Pathway Analysis (IPA) and DAVID revealed that differentially expressed genes in nNCoR and cNCoR clustered into specific functional categories such as *Toll-like signaling pathway*, *Cell Adhesion Molecules* or *negative regulation of apoptosis* (Figure 3B), among others. Moreover, IPA identified NF-κB as the best candidate complex to connect all the observed changes in gene expression between nNCoR and cNCoR (Figure 3C).

### IKK activity regulates NCoR distribution and gene expression in MM cells

Because we here identify NF-κB pathway as the node connecting the transcriptional program altered in cNCoR, and we previously associated the loss of nNCoR in colorectal cancer with increased activity of the NF-κB kinases, IKK, we aimed to investigate whether IKK regulates NCoR distribution in MM cells.

**Figure 4:**
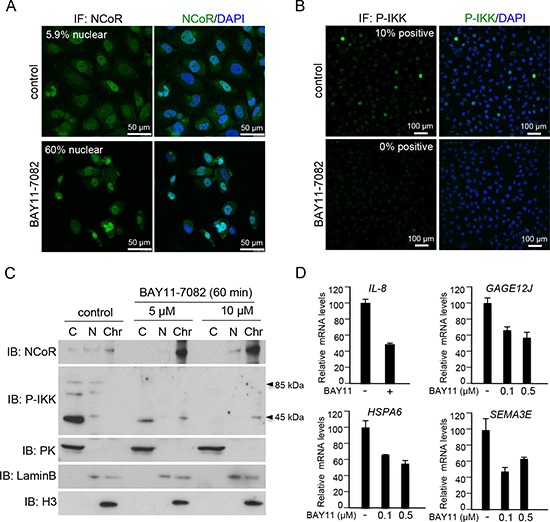
IKK activity regulates NCoR subcellular distribution and function in melanoma cells **(A)** Immunohistochemistry of NCoR in SKMEL-37 melanoma cells untreated or treated for 60 minutes with the IKK inhibitor BAY11-7082. **(B)** Western blot analysis of cytoplasmic, nuclear and chromatin fractions of SKMEL-37 cells, treated with two different concentration of BAY11-7082 (Calbiochem) for 60 minutes. **(C)** qRT-PCR to determine the effect of IKK inhibition by BAY11-7082 (16 hours of treatment) in the levels of various NCoR target genes.

By immunofluorescence, and using the cell lines SKMEL-37 as experimental model, we found that melanoma cells contain variable levels of nNCoR (5.56 ± 2.40%) when grown under standard conditions. Incubation with the IKK inhibitor BAY-11-7082 for 60 minutes resulted in a partial redistribution of cNCoR into the nuclear compartment, up to 59.08 ± 12.19% (Figure 4A). By western blot and IF using a specific antibody detecting active phosphorylated IKK (at serines 180 or 181 for IKKα and IKKβ, respectively), we confirmed that BAY11-7082 effectively blocked IKK activation in these cells (Figure 4B and 4C). Next, we investigated whether nNCoR redistribution, which was achieved after IKK inhibition, was sufficient to revert the basal levels of selected NCoR target genes identified in the microarray screening. By qRT-PCR we found that treatment of SKMEL-37 cells with BAY11-7082 for 60 minutes was sufficient to consistently repress IL8 expression but also other NCoR-regulated genes such as GAGE12J, HSPA6 and SEMA3A (Figure 4D), that are not direct targets of NF-κB.

### NCoR is a putative driver of melanomagenesis

**Figure 5:**
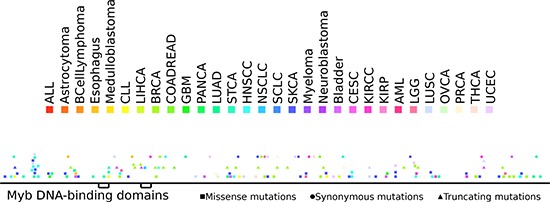
Distribution of somatic mutations along the sequence of the NCoR protein Somatic mutations affecting NCoR across the 7000 samples of the dataset analyzed are color coded according to the tumor type.

NCoR mutations have previously been found in different malignant disorders [15]. Thus, we investigated the possibility that mutations of the NCoR gene also contribute to tumorigenesis in MM. To that effect, we analyzed the existence of NCoR mutations in 369 MM samples from two public studies (see methods for details) and found 23 missense, 4 synonymous and 1 truncating (stop-gain) mutations. Using the IntOGen-mutations platform that distinguishes cancer driver genes from passenger mutations [16], we tested whether NCoR exhibits any signal of positive selection typical of driver genes across the cohorts of MM tumors studied. We found that the mutations present in the Cancer Genome Atlas MM dataset samples as a group were significantly biased towards high functional impact (*p* = 0.002) with respect to background mutations in the same MM samples. Specific driver characteristics of NCoR mutations include the homogeneous distribution along the primary structure of the protein (Figure 5), and a high prevalence of missense and truncating (stop-gain) mutations [17] (Supplementary Table S2).

## DISCUSSION

Malignant melanoma is an aggressive tumor without curative treatment in advance stages. Genetic alterations that activate the MAPK pathway such as mutation on BRAF, N-RAS, and c-Kit, as well as alteration in the p16-CDK4-Rb-ARF-p53 and PTEN-PI3K function are found to promote tumor development through their interaction with other gene regulatory pathways such as NF-κB, and most of this information is currently used for therapy. However, and despite a significant knowledge that has been recently obtained on the molecular alterations leading to MM, the clinical outcome of MM is very variable among patients, and there are no reliable biomarkers (beyond the Breslow index and the staging) that can predict MM behavior and whether particular individuals will benefit from the available treatments. In this context, there is a need of additional molecular clues that allow patient stratification and facilitates future personalized target treatments for MM.

Previous studies from our group identified SMRT and NCoR proteins as key regulators of specific developmental-related genes (such as Hes1 and Herp2) in the intestinal epithelium. Transcriptional control of Hes1 and Herp2 was totally lost in tumors carrying active IKK/NF-κB. We now found that loss of NCoR-mediated gene repression is not restricted tot colorectal cancer but is also found in MM. Most importantly, whereas in colorectal cancer sample alterations of NCoR distribution affected 100% of the studied samples [6], the cytoplasmic accumulation of NCoR in MM correlated with tumor progression, and it is linked with worse clinical outcome. Thus, evaluation of NCoR distribution in melanoma might provide of additional information about prognosis, which is not applicable to colorectal cancer. The differences in nuclear NCoR maintenance or loss between different tumor types might reflect differences in the mechanisms driving tumor transformation, differences in tumor stage at the time of diagnosis, or differential usage of particular signaling pathways (i.e. the NF-κB/IKK pathway).

Since there were no indications about the biologic significance of nNCoR loss in primary MM, we have here performed a high throughput transcriptome analysis of 4 MM samples with nNCoR and 3 MM samples that had lost nNCoR, but they all shared comparable histopathologic characteristics including the tumor stage (all stage II), the anatomic localization, and the distribution of BRAF, NRAS and Kit mutations (see Table 2). Our results led to the identification of a subset of genes that are significantly upregulated in the MM group without nNCoR (group 1), and some of them had been previously associated with cancer progression in other systems. We propose that this here-identified genetic signature could be refined and used as a predictive tool for MM. Not only this, but most of the identified genes participate of pathways or exert specific functions that are targetable, thus we propose that our information should be used to develop and test novel potential therapeutic agents for MM treatment.

On the other hand, it has been previously shown that the NF-κB pathway is active in different MM subtypes. NF-κB is a family of transcription factors that is composed of five different subunit that associate to form homo- and hetero-dimers. The p65/p50 dimer is the most commonly involved in NF-κB signaling, and for this reason detecting nuclear p65-NF-κB is a reasonable approximation of the activation status of the NF-κB pathway. In our MM series, we found that the presence of nuclear p65-NF-κB in MM cells was associated with advanced disease, although the correlation between nuclear p65-NF-κB and cytoplasmic accumulation of NCoR did not reach statistical significance in these tumors. However, since different NF-κB dimers are induced by IKK in a context dependent fashion and because NF-κB regulation is very complex, we directly tested the possibility that NCoR cytoplasmic export in MM was downstream of IKK. With this purpose, we used melanoma cells that showed variable levels of nNCoR. We found that these cells contain active IKK, and that inhibition of IKK by BAY11-7082 reverted nNCoR distribution leading to the transcriptional repression of specific NCoR target genes, which could have clinical or therapeutic implications. Interestingly, it was previously found that nNCoR loss in neuroblastoma is due to a non-canonical activation of the NF-κB pathway by PEDF [18] that could also be explored in MM.

In conclusion, or results indicate that determination of NCoR distribution in MM can be used as a predictive factor, but most importantly as a criterion for patient stratification and identification of individuals that could benefit from future therapies based on NCoR/IKK function modulation. Moreover, differences in the expression profiles associated with the presence or absence of nuclear NCoR in MM might not only give insight in the pathogenic pathways underlying MM development or progression, but also provide a mechanism-driven base for specific therapeutic intervention of selected patients.

## METHODS

### Patients

A total of 63 patients with primary invasive MM, specifically, 38 males and 25 females, with a median age of 69 years (ranging from 31 to 98) were included in the study. Clinical and pathological data have been gathered from a single institution (Parc de Salut MAR from Barcelona, Spain). Cases analyzed in this study include all patients with invasive MM that were recruited during the period 2000–2009 from Parc de Salut Mar-Biobank, only excluding non-conventional histological variants or doubtful cases of melanocytic proliferations with unknown significance. Cases were not selected on the basis to any specific clinical or histopathological criteria.

Forty-six patients presented a Breslow index > 1 mm (mean: 2.24 mm; median 1.4 mm). Fourteen tumors were ulcerated and 31 showed a mitotic index > 1 mitoses/mm^2^, 18 cases presented with 1 mitosis/mm^2^ and no mitoses were seen in the remaining 14 cases. Patients were periodically controlled for a median follow-up of 59.4 months. Forty-nine patients were classified in stage I–II and 14 were in stages III–IV at diagnosis, and from those in which sentinel lymph node biopsies were performed (32 out of 63 patients), 19% (6 patients) presented micrometastases. During the follow-up period, 48 patients did not progress in the disease from the initial stage (IA–IIIA), whereas 15 cases (24%) progressed to more advanced stages. These clinical data are representative of an average MM population.

### Immunohistochemistry (IHC) and immunofluorescence (IF)

IHC and IF analysis of the MM samples and cell lines was performed using the following antibodies: Goat polyclonal anti-NCoR (sc-C20) and p65-NF-κB (sc-372) from Santa Cruz biotechnology inc. Antigen retrieval was achieved by boiling the samples for 10 minutes in Tris/EDTA buffer at pH 9.0. Tissue sections were incubated overnight with the corresponding primary antibody (in PBS plus 0.1% BSA), and then extensively washed and incubated with the secondary biotin-labeled antibodies (1:200) at room temperature for 30 minutes. Immunoreactivity was revealed using a streptavidin-biotin-peroxidase complex (sABC-HRP, DakoCytomation K0377; 1:100; DAKO) or Alcaline phosphatase, and developed with diaminobenzidine (DAB) solution (Sigma–Aldrich, Zwijndrecht, The Netherlands) or Fast Red TR (Sigma), respectively. All sections were then counterstained with hematoxylin, and Melan-A was used to confirm the neoplastic (melanocytic) nature of the cells stained for NCoR and p65-NF-κB. Cells were counted directly under a 40× magnification and the percentage of tumor cells that exhibited positive immunoreactivity was determined. At least 100 neoplastic cells were counted for each sample. The mean percentage obtained from 3 independent reads by 2 pathologists was recorded. Nuclear and cytoplasmic staining was evaluated separately. Cell staining scores were analyzed as percentages of cells in each degree of staining. For nuclear NCoR determination only the number (percentage) of nuclei with moderate to intense expression was considered. Intense nuclear staining was considered when it was comparable to the non-tumor samples used as controls. Moderate refers to positivity that was less intense than normal controls. No or light expression was considered when we did not detect a consistent staining at the 40× magnification. Staining scores were compared using Student's *t*-test or analysis of variance (ANOVA) and non-parametric U-Mann–Whitney was employed when data did not meet the conditions of application of these tests. Likewise comparisons in which subgroups did not reach the minimum sample size Bonferroni corrections were used. Kaplan Meier survival curves were calculated using the SPSS program. The morphological variables and IHC data were recorded and included the histopathological subtype of MM, measurements of invasion (Breslow index and Clark level), the presence of ulceration, mitotic rate, features of regression, intratumor inflammatory infiltrate, the presence of microsatellitosis and vascular invasion. In addition, 40 benign melanocytic lesions (intradermal, junctional and dysplastic melanocytic nevi) were used as controls. Sample size for comparing two means with unilateral contrast, being the percentage of positively stained melanocytes the main variable, was calculated according the algorithm, *n* = ((za + z2b)^2 *2*s ^2)/Dm^. We used the semi-quantitative Histoscore index: (0x%) + (1x%) + (2x%) + (3x%) to perform parametric statistical tests to determine the association between nuclear NCoR and p65 stainings.

### WB and IKK inhibitors

Western blot experiments were performed using standard methods. The α-NCoR antibody was from Abcam (ab58396), α-P-IKKα-Ser180/IKKβ-Ser181 (sc-23470) and α-Lamin B (sc-6216) were from Santa Cruz Biotechnology, and α-tubulin from Sigma. BAY11-7082 was purchased from Calbiochem (7082) and used at the indicated concentrations.

### Obtaining and processing somatic mutations datasets

We obtained 51 somatic mutations datasets from the International Cancer Genome Consortium (ICGC), the Cancer Genome Atlas (TCGA), and literature searches, analogously as described [16]. Nine ICGC datasets were downloaded directly from the Data Coordination Centre (DCC) Biomart [19]. Nineteen TCGA datasets were downloaded from the Synapse platform (syn1729383) as MAF files. We obtained thirteen additional datasets from a comprehensive recent study of tumor mutational signatures [20]. Finally, a manual PubMed search allowed us to identify ten other somatic mutations datasets that had been produced by research groups outside these large initiatives. Among these 51 datasets, there were two MM studies, one carried out by the Broad Institute (and obtained via [20]) and a second one developed within the TCGA (downloaded from Synapse) totaling 369 samples.

### Running the IntOGen-mutations pipeline

The ICGC and mutational signatures datasets were obtained already in the tab-separated format accepted by the pipeline. The MAF files obtained from synapse were parsed and transformed to this tab-separated format. We manually parsed supplementary files of the papers reporting individual studies to extract the lists of somatic mutations detected across tumor samples and then transformed them into tab-separated files. The first part of the IntOGen-mutations pipeline [16] assesses the potential functional impact of somatic mutations detected across the cohort of tumor samples. The Ensembl Variant Effect Predictor (VEP, v.70 script [21] and pre-computed cache files, downloaded from the Ensembl ftp site (ftp://ftp.ensembl.org/pub/), are used to determine the consequences of somatic mutations to annotated protein-coding genes. We employed the results of this first part to compute the number of samples with mutations in NCoR in different datasets –subsequently aggregated per tumor type–, as well as their consequence types. The pipeline also obtains SIFT [22] and PolyPhen2 [23] functional impact scores from the VEP. Pre-computed Mutation Assessor [24] functional impact obtained from its author's webserver (www.mutationassessor.org) during the installation of the pipeline are queried locally during execution. The pipeline implements an expression filter to disregard genes that are not expressed across the tumor types analyzed.

The Oncodrive FM approach has also been described elsewhere [17]. Briefly, Oncodrive FM receives as input the list of synonymous, stop-gained, stop-lost, non-synonymous and frameshift-indel mutations and their corresponding SIFT, PolyPhen2 and Mutation Assessor scores. Then, it assesses whether any gene shows a trend toward the accumulation of mutations with high functional impact as compared to the background distribution of these functional impact scores in all mutations detected across the cohort of tumor samples (FM bias). For each functional impact score method included in the pipeline, the method produces an empirical *p*-value that evaluates this FM bias. These three *p*-values are subsequently combined using Fisher's approach to produce one integrated *p*-value for each gene. To account for possible non-dependence between the three *p*-values included in the combination, the IntOGen-mutations web discovery tool considers as significant those with a false discovery rate (FDR) below 0.01. We checked specifically whether NCoR exhibits the FM bias in any mutational dataset. (When more than one dataset of the same tumor type was available, we retained only the most significant *q*-value: see Supplementary Table S2)

### Gene ontology analysis of differentially expressed genes

Overrepresentation analysis of Gene Ontology (GO) categories and pathways was used to explore the functions associated with NCoR regulated genes in MM. Gene set enrichment analysis (GSEA) identifies gene sets consisting of co-regulated genes; Gene Ontology (GO) gene sets are based on ontologies and do not necessarily comprise co-regulated genes. Gene sets collected from various sources such as online pathway databases, publications in PubMed, and knowledge of domain experts. The gene set page for each gene set lists its source. Computational gene sets defined by mining large collections of cancer-oriented microarray data. Gene sets are named by GO term and contain genes annotated by that term. (http://www.broadinstitute.org/gsea/msigdb/collections.jsp#C2); IPA: (http://www.ingenuity.com).

### Ethical considerations

The approval for the study was provided by the Comitè Ètic d’ Investigació Clínica from Institut Municipal d’Assistència Sanitària (CEIC-PSMAR) and written informed consent was obtained from all patients according to the National and International guidelines (code of ethics, Declaration of Helsinki) and the legal regulations on data privacy (Law 15/1999 of 13 December on the Protection of Personal Data) were considered. All samples stored in the tumor bank (Hospital del Mar in Barcelona, Spain).

### Statistical analysis

Non-parametric Kruskal–Wallis test and Mann–Whitney *U* test were used to determine the association between NCoR and various categorical clinicopathologic parameters. To assess the relationship between NCoR with Breslow index and mitotic rate, Spearman's correlation coefficient was performed. Kaplan–Meier method with a Log-Rank test was used to compare overall survival, disease specific survival and disease free survival among groups. *P* values less than 0.05 were considered as statistically significant. Analysis performed with SPSS 18.0 (IBM Corp.).

## SUPPLEMENTARY METHODS


