# Supplementary material for: Cytoplasmic accumulation of NCoR in malignant melanoma: consequences of altered gene repression and prognostic significance
Source: Oncotarget. 2015 Mar 19;6(11):9284–94. doi: 10.18632/oncotarget.3252 (PMC4496217; doi:10.18632/oncotarget.3252)
Supplement: Supplementary file 1 [file oncotarget-06-9284-s001.pdf]

SUPPLEMENTARY METHODS

Microarray sample processing

Frozen samples in which malignant cells constituted 80% or more of the total number of infiltrating cells were selected for expression microarray analyses. Samples and microarrays were processed according to the manufacturer's protocol (available from Affymetrix, Santa Clara, CA). The array images were quantified using the MicroArray Suite (MAS) v 5.0 software (Affymetrix). The average fluorescence intensity was determined for each microarray, and then the output of each experiment was globally scaled to a target value of 200. Normalization of the data was performed using variant stability and normalization (VSN, Stanford, CA), part of the R statistical software package (available at www.bioconductor.org). Gene expression patterns were further analyzed using Spotfire DecisionSite (Spotfire, Goteborg, Sweden) and Statistical Analysis of Microarrays (SAM, Stanford, CA). First, unsupervised hierarchical clustering (i.e. based on the expression of all genes present at the microarray) was performed using Spotfire DecisionSite. Statistical Analysis of Microarrays (SAM) was applied to compare the gene expression pattern of MM samples with nuclear or cytoplasmic NCoR. A false discovery rate of less than 1.0 was chosen to select genes that were significantly up- or down-regulated in both groups. Gene expression data were transformed into z-scores as described previously, and obtained output was visualized using Spotfire DecisionSite.

SUPPLEMENTARY TABLES

Supplementary Table S1: Genes up-regulated in MM with loss of nuclear NCoR

Symbol	Description	Log FC	Ave Expr	*P* Value
**GAGE12J**	G antigen 12J	–2.93	4.37	0
**IL8**	Interleukin 8	–2.21	6.05	0.03
**HSPA1B**	Heat shock 70kDa protein 1B	–2.16	6.95	0
**C21orf94**	Chromosome 21 open reading frame 94	–2.08	2.76	0.03
**TFPI2**	Tissue factor pathway inhibitor 2	–2	4.08	0.05
**SEMA3E**	Semaphorin 3E	–1.99	3.86	0.02
**HSPA6**	Heat shock 70kDa protein 6 (HSP70B')	–1.93	5.66	<0.001
**MAGEC2**	Melanoma antigen family C. 2	–1.87	3.97	0
**EYA4**	Eyes absent homolog 4 (Drosophila)	–1.85	4.69	0
**SERPINE1**	Serpin peptidase inhibitor member 1	–1.81	6.48	0.02
**TIMP3**	TIMP metallopeptidase inhibitor 3	–1.74	9.34	0.03
**MCAM**	Melanoma cell adhesion molecule	–1.72	6.06	0
**FAM40B**	Family with sequence similarity 40. member B	–1.69	4.94	0
**SNORD14C**	Small nucleolar RNA. C/D box 14C	–1.66	6.04	0
**NA**	ncRNA	–1.64	3.39	0
**SNORD14E**	Small nucleolar RNA. C/D box 14E	–1.63	4.7	0.01
**PPP1R9A**	Protein phosphatase 1 regulatory subunit 9A	–1.58	5.68	0.01
**C7orf68**	Chromosome 7 open reading frame 68	–1.57	5.97	0
**GEM**	GTP binding protein overexpressed in skeletal muscle	–1.57	5.52	0.01
**ATP6V0A4**	ATPase. H+ transporting. lysosomal V0 subunit a4	–1.57	4.66	0.01

(*Continued*)

Symbol	Description	Log FC	Ave Expr	*P* Value
**LOC388022**	Hypothetical LOC388022	–1.57	6.03	0.01
**SOX6**	SRY (sex determining region Y)-box 6	–1.51	5.82	0.01
**ACSS3**	Acyl-CoA synthetase short-chain family member 3	–1.51	4.67	0.04
**PYGL**	Phosphorylase. glycogen. liver	–1.48	8.21	0.01
**SNORD43**	Small nucleolar RNA. C/D box 43	–1.47	6.3	0
**TREM1**	Triggering receptor expressed on myeloid cells 1	–1.46	4.47	0.02
**GAGE12B**	G antigen 12B	–1.43	5.68	0.05
**LY96**	Lymphocyte antigen 96	–1.42	4.26	0.05
**HK2**	Hexokinase 2	–1.41	6.68	0.01
**SNORD3A**	Small nucleolar RNA. C/D box 3A	–1.41	9.47	0.01
**FOS**	FBJ murine osteosarcoma viral oncogene homolog	–1.4	8.78	0.05
**STK32B**	Serine/threonine kinase 32B	–1.38	5.94	0
**KCNH1**	Potassium voltage-gated channel Subfamily member 1	–1.36	4.43	0.03
**TUBA8**	Tubulin. alpha 8	–1.36	5.09	0.03
**ACTN2**	Actinin. alpha 2	–1.35	4.28	0
**DNAJB1**	DNAJ (Hsp40) homolog. subfamily B. member 1	–1.35	7.17	0
**SLC5A4**	Solute carrier family 5 member 4	–1.35	4.01	0
**IGF2BP1**	Insulin-like growth factor 2 mRNA binding protein 1	–1.33	4.39	0.01
**HMOX1**	Heme oxygenase (decycling) 1	–1.32	7.22	0.01
**SLC11A1**	Solute carrier family 11 member 1	–1.32	4.78	0.01
**CPM**	Carboxypeptidase M	–1.32	6.26	0.01
**MMP3**	Matrix metallopeptidase 3 (stromelysin 1. progelatinase)	–1.32	4.71	0.04
**PLIN2**	Perilipin 2	–1.31	6.19	0.01
**SLC5A6**	Solute carrier family 5 member 6	–1.29	5.59	0.01
**CSF3R**	Colony stimulating factor 3 receptor (granulocyte)	–1.29	4.63	0.01
**LDHC**	Lactate dehydrogenase C	–1.29	2.96	0.04
**NA**	ncRNA	–1.28	3.73	0
**DAAM2**	Dishevelled associated activator of morphogenesis 2	–1.28	5.66	0
**HMGA2**	High mobility group AT-hook 2	–1.28	4.72	0.02
**SLC7A11**	Solute carrier family 7 member 11	–1.27	4.44	0
**SNORD74**	Small nucleolar RNA. C/D box 74	–1.27	6.53	0.01
**NA**	ncRNA	–1.27	3.23	0.01
**ZFAND2A**	Zinc finger. AN1-type domain 2A	–1.26	7.34	0
**PPP1R15A**	Protein phosphatase 1 regulatory subunit 15A	–1.26	7.12	0
**STARD13**	STAR-related lipid transfer domain containing 13	–1.26	5.47	0.03
**NA**	ncRNA	–1.25	4.42	0.02
**DZIP1**	DAZ interacting protein 1	–1.25	5.33	0.02

(*Continued*)

Symbol	Description	Log FC	Ave Expr	*P* Value
**SNORD58A**	Small nucleolar RNA. C/D box 58A	–1.24	4.89	0.02
**NA**	ncRNA	–1.23	4.41	0.01
**IFRD1**	Interferon-related developmental regulator 1	–1.21	6.88	0
**MAGEC1**	Melanoma antigen family C. 1	–1.21	3.72	0.01
**SNORD34**	Small nucleolar RNA. C/D box 34	–1.2	4.99	0
**MAGEA3**	Melanoma antigen family A. 3	–1.2	4.6	0
**LRRFIP1**	Leucine rich repeat (in FLII) interacting protein 1	–1.2	5.63	0.02

Supplementary Table S2: Mutational analysis of NCoR in different types of tumors

Tumor type	Tumor acronym	Samples	Synonymous	Mutations Missense	Truncating	Fmbias(q value)*
Acute lymphoid leukemia	ALL	122	0	0	0	-
Pylocitic Astrocytoma	Astrocytoma	101	0	0	0	-
Diffuse B Cell lymphoma	BCell Lymphoma	23	0	0	0	-
Esophagus carcinoma	Esophagus	146	2	1	0	-
Medulloblastoma	Medulloblastoma	213	0	0	0	-
Chronic lymphocytic leukemia	CLL	452	0	4	0	0,008
Liver hepatocarcinoma	LIHCA	90	0	1	0	-
Breast carcinoma	BRCA	1148	2	13	16	0
Colorectal adenocarcinoma	COADREAD	229	1	0	1	-
Glioblastoma multiforme	GBM	379	0	2	0	-
Pancreas carcinoma	PANCA	248	0	0	0	-
Lung adenocarcinoma	LUAD	391	2	7	0	1
Stomach carcinoma	STCA	161	2	6	1	0,42
Head and neck squamous cell carcinoma	HNSCC	375	1	14	1	0,03
Non small cell lung cancer	NSCLC	31	0	0	1	-
Small cell lung cancer	SCLC	69	0	0	0	-
Cutaneous melanoma	SKCM	369	4	23	1	0,002
Myeloma	Myeloma	69	0	0	0	-
Neuroblastoma	Neuroblastoma	210	0	0	0	-

(*Continued*)

Tumor type	Tumor acronym	Samples	Synonymous	Mutations Missense	Truncating	Fmbias(q value)*
Bladder carcinoma	Bladder	96	1	4	3	0,16
Cervical squamous cell carcinoma	CESC	39	0	0	0	-
Kidney renal clear cell carcinoma	KIRCC	417	3	3	0	1
Kidney papillary carcinoma	KIRP	100	0	0	0	-
Acute myeloid leukemia	AML	196	0	0	0	-
Lower grade glioma	LGG	169	1	0	0	-
Lung squamous cell carcinoma	LUSC	174	3	5	2	1
Ovarian serous adenocarcinoma	OVCA	316	0	1	0	-
Prostate carcinoma	PRCA	243	1	2	1	0,75
Thyroid carcinoma	THCA	322	2	0	0	-
Uterine corpus endometrioid carcinoma	UCEC	230	2	3	0	1

*Dataset with lowest q value in a tumor type
